# Trends in SARS-CoV-2 seroprevalence among pregnant women attending first antenatal care visits in Zambia: A repeated cross-sectional survey, 2021–2022

**DOI:** 10.1371/journal.pgph.0003073

**Published:** 2024-04-03

**Authors:** Elizabeth Heilmann, Tannia Tembo, Sombo Fwoloshi, Bupe Kabamba, Felix Chilambe, Kalubi Kalenga, Mpanji Siwingwa, Conceptor Mulube, Victoria Seffren, Carolyn Bolton-Moore, John Simwanza, Samuel Yingst, Ruchi Yadav, Eric Rogier, Andrew F. Auld, Simon Agolory, Muzala Kapina, Julie R. Gutman, Theodora Savory, Chabu Kangale, Lloyd B. Mulenga, Izukanji Sikazwe, Jonas Z. Hines

**Affiliations:** 1 Public Health Institute, Oakland, California, United States of America; 2 Division of Global HIV and Tuberculosis, U.S. Centers for Disease Control and Prevention, Lusaka, Zambia; 3 Centre for Infectious Disease Research in Zambia, Lusaka, Zambia; 4 Division of Infectious Diseases, Ministry of Health, Lusaka, Zambia; 5 PATH, Lusaka, Zambia; 6 Adult Centre of Excellence, University Teaching Hospital, Lusaka, Zambia; 7 Division of Parasitic Diseases and Malaria, U.S. Centers for Disease Control and Prevention, Atlanta, Georgia, United States of America; 8 Surveillance and Disease Intelligence, Zambia National Public Health Institute, Lusaka, Zambia; Fundacao Oswaldo Cruz, BRAZIL

## Abstract

SARS-CoV-2 serosurveys help estimate the extent of transmission and guide the allocation of COVID-19 vaccines. We measured SARS-CoV-2 seroprevalence among women attending ANC clinics to assess exposure trends over time in Zambia. We conducted repeated cross-sectional SARS-CoV-2 seroprevalence surveys among pregnant women aged 15–49 years attending their first ANC visits in four districts of Zambia (two urban and two rural) during September 2021-September 2022. Serologic testing was done using a multiplex bead assay which detects IgG antibodies to the nucleocapsid protein and the spike protein receptor-binding domain (RBD). We calculated monthly SARS-CoV-2 seroprevalence by district. We also categorized seropositive results as infection alone, infection and vaccination, or vaccination alone based on anti-RBD and anti-nucleocapsid test results and self-reported COVID-19 vaccination status (vaccinated was having received ≥1 dose). Among 8,304 participants, 5,296 (63.8%) were cumulatively seropositive for SARS-CoV-2 antibodies from September 2021 through September 2022. SARS-CoV-2 seroprevalence primarily increased from September 2021 to September 2022 in three districts (Lusaka: 61.8–100.0%, Chongwe: 39.6–94.7%, Chipata: 56.5–95.0%), but in Chadiza, seroprevalence increased from 27.8% in September 2021 to 77.2% in April 2022 before gradually dropping to 56.6% in July 2022. Among 5,906 participants with a valid COVID-19 vaccination status, infection alone accounted for antibody responses in 77.7% (4,590) of participants. Most women attending ANC had evidence of prior SARS-CoV-2 infection and most SARS-CoV-2 seropositivity was infection-induced. Capturing COVID-19 vaccination status and using a multiplex bead assay with anti-nucleocapsid and anti-RBD targets facilitated distinguishing infection-induced versus vaccine-induced antibody responses during a period of increasing COVID-19 vaccine coverage in Zambia. Declining seroprevalence in Chadiza may indicate waning antibodies and a need for booster vaccines. ANC clinics have a potential role in ongoing SARS-CoV-2 serosurveillance and can continue to provide insights into SARS-CoV-2 antibody dynamics to inform near real-time public health responses.

## Introduction

The first COVID-19 case was detected in Zambia on March 18, 2020 [1]. Since then, the Zambia National Public Health Institute (ZNPHI) has reported nearly 350,000 COVID-19 cases over four main epidemic waves from 2020 through 2023 in Zambia [2]. Confirmed case counts underreport the true extent of SARS-CoV-2 infections due to the high proportion of subclinical infections, limited testing supplies, and surveillance gaps. SARS-CoV-2 seroprevalence studies can help bridge the gap in understanding SARS-CoV-2 transmission. In Zambia, a survey of six districts found a pooled prevalence of 10.6% in July 2020 and estimated that 92 infections occurred for every reported case [3]. Another study conducted in a peri-urban district in February 2021 measured a seroprevalence of 33.7% [4]. No studies have been published measuring SARS-CoV-2 seroprevalence in Zambia following the Delta and Omicron waves.

Repeated cross-sectional surveys leveraging existing healthcare platforms can help monitor trends in seroprevalence during ongoing transmission while minimizing implementation costs associated with large-scale household surveys. Antenatal care (ANC) clinics have been utilized in several countries in Africa to monitor SARS-CoV-2 seroprevalence in a healthy population of women accessing healthcare services [5–9]. In Ethiopia, SARS-CoV-2 surveillance conducted in ANC clinics between April 2020 and March 2021 first detected SARS-CoV-2 antibodies in June 2020 and peaked at 11.8% in February 2021 [10]. Repeated cross-sectional serosurveys in ANC clinics in Kenya found that most women were seropositive for SARS-CoV-2 antibodies by October 2021 [11].

Early SARS-CoV-2 serosurveys measured antibodies to estimate total infections, but COVID-19 vaccine rollout now clouds seroprevalence interpretations. Detectable antibodies may be the result of SARS-CoV-2 infection, COVID-19 vaccination, or both. Serologic assays with nucleocapsid and spike targets in combination with COVID-19 vaccination history can distinguish these groups in countries where most vaccines administered target the spike protein, such as in Zambia [12]. COVID-19 vaccines first became publicly available in Zambia in April 2021 [13]. After a slow rollout initially, the Ministry of Health announced achieving 70% coverage of the targeted population on November 1, 2022 [14]. Most COVID-19 vaccine doses received and distributed in Zambia have been the spike-targeting Janssen (61%) and Pfizer-BioNTech (20%) vaccines [13].

In Zambia, 97% of women attend at least one ANC visit during pregnancy, and antenatal attendees have long served as a sentinel population for HIV, syphilis, and malaria surveillance [15–17]. Whether this platform can be leveraged to monitor COVID-19 has yet to be determined. We aimed to estimate monthly seroprevalence of SARS-CoV-2 antibodies among pregnant women attending first antenatal care visits at health facilities in four districts of Zambia from September 2021 through September 2022.

## Methods

### Study setting and population

We did an implementation research study of repeated cross-sectional SARS-CoV-2 seroprevalence surveys among women attending first ANC visits in four districts (Chadiza, Chipata, Chongwe, and Lusaka) in Zambia from September 2021 to September 2022. Data collection began after Zambia’s third epidemic wave caused by the Delta variant (May-August 2021) and encompassed the fourth COVID-19 wave driven by the Omicron variant (December 2021-March 2022). The four districts were purposefully selected to capture a range of urban (Chipata and Lusaka) and rural (Chadiza and Chongwe) communities and districts with points-of-entry into Zambia (e.g., international airport in Lusaka and land borders with Malawi and Mozambique in Chipata and Chadiza, respectively).

A sample size of 200 women per district per month was selected based on available resources and an acceptable margin of error for the expected range of SARS-CoV-2 seroprevalence estimates over the study period. Ten facilities per district were randomly selected using probability proportional to the average number of ANC enrollments per month among health facilities reporting an average of at least 19 antenatal patient enrollments per month in 2020. Each facility in Chipata, Chongwe, and Lusaka districts enrolled up to 20 participants per month among pregnant women aged 15–49 years attending their first ANC visits. A health worker provided information on the study during group counseling sessions when the health facility provided routine first-time ANC services. Pregnant women willing to participate were screened for eligibility and, if consenting, were enrolled in the study until 20 participants were recruited per facility each month.

In Chadiza District, all women attending the first ANC were approached for participation as part of a larger malaria surveillance pilot project, and up to 200 samples were selected post-enrollment for SARS-CoV-2 serologic testing per month [18]. For months with >200 participants in Chadiza, within each month, records were ordered by facility and interview date and the first 20 participants’ specimens were tested for SARS-CoV-2. If a facility had fewer than 20 participants in a month, specimens from other sites in Chadiza were selected and tested based on interview date until the sample size of 200 was reached. SARS-CoV-2 surveillance in Chadiza goes until July 2022 (instead of September 2022) when the malaria surveillance project ended.

At all sites, women were screened for acute COVID-19 prior to enrollment, and those exhibiting symptoms were referred for COVID-19 testing and case management per government protocols and were excluded from the study.

### Data collection and sample processing

Participants were administered an electronic questionnaire on tablets using the Open Data Kit platform (Get ODK Inc., San Diego, CA, USA) to gather demographic data, SARS-CoV-2 exposures, COVID-19 vaccination, and routine ANC test results (i.e., HIV, syphilis, hepatitis, and malaria). Possible SARS-CoV-2 exposures included participants or households prior positive SARS-CoV-2 test and sustained close contact with a suspected or known COVID-19 case. COVID-19 vaccination and prior COVID-19 statuses were self-reported. Study staff attempted to verify COVID-19 vaccination information from vaccination cards if participants had them available on the day of their ANC visit. From February 2022 onward, participants self-reported inter-district and international travel, frequency of visits to churches, funerals or weddings, markets, and indoor dining, and use of public transportation and face masks to provide a general sense of COVID-19 risk behaviors.

Whole blood was collected on filter paper as dried blood spots (DBS) using the same finger prick or venipuncture from routine ANC testing. HIV, syphilis, hepatitis B, and malaria tests were conducted according to national guidelines, subject to the availability of test kits. Chipata, Chongwe, and Lusaka district specimens were transported to a central laboratory for testing at the University Teaching Hospital (Lusaka, Zambia) and Chadiza District specimens were transported for testing at PATH laboratory (Lusaka, Zambia).

Serologic testing was done using the FlexImmArray SARS-CoV-2 Human IgG Antibody Test (Tetracore Inc, Rockville, MD, USA) on the MAGPIX platform (Luminex Corp, Austin, TX, USA) according to manufacturer instructions with DBS preparation as described by Tartof et al. [19, 20]. The assay was verified in-country in conjunction with the US CDC prior to its use. This multiplex bead assay has three SARS-CoV-2 targets: nucleocapsid (N) protein, the receptor binding domain (RBD) of the spike-1 protein, and a N-RBD fusion protein. Signal ratios were calculated for each target as the median fluorescent intensity of the protein divided by the average calibrator median fluorescent intensity from each plate. Samples with signal ratios ≥1.2 for all three targets were considered SARS-CoV-2 IgG positive for past SARS-CoV-2 exposure, and samples with a signal ratio ≤0.9 for any target were considered SARS-CoV-2 IgG negative. Signal ratios >0.9 and <1.2 were considered indeterminate and the samples were re-run. Valid results of the second test were retained in the final dataset. Samples with indeterminate results after re-testing were categorized as SARS-CoV-2 IgG negative. Samples with persistent quality control issues were removed from the final dataset.

### Statistical analysis

SARS-CoV-2 seroprevalence was estimated as the number of participants with a positive test result divided by the total number of participants with a valid test result by month and district. Seroprevalence was adjusted based on the assay sensitivity (89.8%) and specificity (100.0%) from an independent test [21]. Trend analysis was conducted to compare SARS-CoV-2 seroprevalence with district-specific COVID-19 confirmed cases provided by ZNPHI [2].

In a sub-analysis, target-specific assay results and COVID-19 vaccination status were analyzed to group antibody responses into no evident response (anti-RBD negative), infection only (anti-RBD positive, unvaccinated), vaccination only (anti-RBD positive, vaccinated, anti-N negative), or vaccination and infection (anti-RBD positive, vaccinated, anti-N positive) based off the decision tree developed by Duarte et al. [12]. Antibody responses in participants who received an unknown or inactivated virus vaccine were classified as indeterminate. Wilcoxon rank sum tests were used to compare signal ratios for each assay target by antibody response category. Logistic regression controlling for enrollment month was used to identify factors associated with seropositivity and calculate adjusted odds ratios. Sub-analyses were conducted among participants who reported prior confirmed COVID-19 and those who had received a COVID-19 vaccine.

District-level populations exposed to SARS-CoV-2 were calculated using 2022 Census population projection estimates from the Zambia Statistics Agency and peak SARS-CoV-2 seroprevalence estimates during data collection [22]. These peak estimates of persons exposed to SARS-CoV-2 were compared to the total number of reported COVID-19 cases at the end of the study to estimate the ratio of reported cases to total SARS-CoV-2 infections in each district.

All analyses were conducted using R version 4.2.1 (R Foundation for Statistical Computing, Vienna, Austria).

This study was authorized by the National Health Research Authority (NHRA) through an expedited review of COVID-19 related studies. For Chadiza District, the SARS-CoV-2 data collection and serologic testing were reviewed and approved by the University of Zambia Biomedical Research Ethics Committee as an amendment to a pre-existing malaria surveillance protocol. This activity was reviewed by CDC and was conducted consistent with applicable federal law and CDC policy (See e.g., 45 C.F.R. part 46.102(l)(2), 21 C.F.R. part 56; 42U.S.C. §241(d); 5 U.S.C. §552a;44 U.S.C. §3501 et seq). All methods were carried out in accordance with relevant guidelines and regulations. Written informed consent was obtained for all participants before enrollment. Pregnant women under 18 years are considered emancipated minors in Zambia for the purpose of providing their own informed consent.

## Results

From September 2021 through September 2022, 9,221 pregnant women attending their first ANC visits were enrolled in the study. A total of 8,558 (92.8%) samples were tested; among those, 8,304 (97.0%) valid SARS-CoV-2 antibody results were matched to participant questionnaires (Chadiza: 1,616, Chipata: 2,099, Chongwe: 2,441, Lusaka: 2,148). Two-hundred and fifty-four specimens could not be linked to questionnaire data, so were not included in the analysis.

The median age of participants was 25 years (interquartile range [IQR]: 20–30) (Table 1). The majority (57.9%) of participants achieved primary education or less and most were unemployed (40.2%) or farmers (24.9%). Only 2.2% of women reported having COVID-19 prior to study enrollment, 3.3% reported confirmed COVID-19 in the household, and 3.9% reported close contact with a confirmed or suspected COVID-19 case outside the household. About half of the participants responded that they wore a mask all or most of the time in public (49.5%) or were observed to be wearing a face mask properly during the ANC visit (49.3%). Vaccination coverage of ≥1 COVID-19 vaccine doses increased from 4.4% in September 2021 to 26.6% in September 2022. Most (87.4%) vaccinated women were considered fully vaccinated with a primary series, most having received the Janssen vaccine. Routine ANC testing for syphilis and hepatitis B was reported in less than half of the participants (40.2% and 11.7%, respectively), but most (96.7%) women were tested for HIV or knew their HIV status prior to the visit and 8.8% were positive.

**Table 1:** Participant demographics, SARS-CoV-2 exposures and risk behaviors, and ANC test results.

	Chadiza		Chipata		Chongwe		Lusaka		Total	
	n = 1616		n = 2099		n = 2441		n = 2148		N = 8304	
	n	(%)	n	(%)	n	(%)	n	(%)	n	(%)
Age, median (IQR)	22	(19–28)	25	(21–30)	24	(20–30)	26	(22–30)	25	(20–30)
15–19	438	(27.1)	369	(17.6)	518	(21.2)	210	(9.8)	1535	(18.5)
20–29	760	(47.0)	1190	(56.7)	1300	(53.3)	1297	(60.4)	4547	(54.8)
30–39	251	(15.5)	481	(22.9)	531	(21.8)	576	(26.8)	1839	(22.1)
40–49	26	(1.6)	43	(2.0)	90	(3.7)	57	(2.7)	216	(2.6)
Unknown	141	(8.7)	16	(0.8)	2	(0.1)	8	(0.4)	167	(2.0)
Education										
No education	377	(23.3)	171	(8.1)	77	(3.2)	31	(1.4)	656	(7.9)
Some primary	779	(48.2)	518	(24.7)	809	(33.1)	413	(19.2)	2519	(30.3)
Completed primary	133	(8.2)	553	(26.3)	608	(24.9)	337	(15.7)	1631	(19.6)
Some secondary	200	(12.4)	612	(29.2)	732	(30.0)	805	(37.5)	2349	(28.3)
Completed secondary or higher	123	(7.6)	240	(11.4)	215	(8.8)	559	(26.0)	1137	(13.7)
Unknown	4	(0.2)	5	(0.2)	0	(0.0)	3	(0.1)	12	(0.1)
Occupation										
Farmer	1383	(85.6)	443	(21.1)	602	(24.7)	10	(0.5)	2438	(24.9)
Daily laborer/pieceworker	8	(0.5)	78	(3.7)	325	(13.3)	64	(3.0)	475	(5.7)
Merchant/shop owner	12	(0.7)	56	(2.7)	55	(2.3)	108	(5.0)	231	(2.8)
Health worker	8	(0.5)	16	(0.8)	6	(0.2)	13	(0.6)	43	(0.5)
Government employee/civil servant	27	(1.7)	47	(2.2)	17	(0.7)	88	(4.1)	179	(2.2)
Private sector employee	6	(0.4)	31	(1.5)	25	(1.0)	145	(6.8)	207	(2.5)
Hawker/market seller	14	(0.9)	142	(6.8)	164	(6.7)	241	(11.2)	561	(6.8)
Student	89	(5.5)	95	(4.5)	79	(3.2)	111	(5.2)	374	(4.5)
Unemployed	63	(3.9)	996	(47.5)	1088	(44.6)	1195	(55.6)	3342	(40.2)
Other occupation	1	(0.1)	162	(7.7)	54	(2.2)	162	(7.5)	379	(4.6)
Unknown	5	(0.3)	33	(1.6)	26	(1.1)	11	(0.5)	75	(0.9)
Prior COVID-19										
No	1565	(96.8)	2031	(96.8)	2375	(97.3)	2054	(95.6)	8025	(96.8)
Yes	42	(2.6)	42	(2.0)	31	(1.3)	64	(3.0)	179	(2.2)
Unknown	9	(0.6)	26	(1.2)	35	(1.4)	30	(1.4)	100	(1.2)
Household COVID-19										
No	1573	(97.3)	2014	(96.0)	2276	(93.2)	1983	(92.3)	7846	(94.5)
Yes	36	(2.2)	49	(2.3)	48	(2.0)	137	(6.4)	270	(3.3)
Unknown	7	(0.4)	36	(1.7)	117	(4.8)	28	(1.3)	188	(2.3)
Close COVID-19 contact^†^										
No	1439	(89.0)	1953	(93.0)	1993	(81.6)	1862	(86.7)	7247	(87.3)
Yes	30	(1.9)	52	(2.5)	62	(2.5)	176	(8.2)	320	(3.9)
Unknown	147	(9.1)	94	(4.5)	386	(15.8)	110	(5.1)	737	(8.9)
COVID-19 vaccination^‡^										
No	1089	(67.4)	1633	(77.8)	1925	(78.9)	1904	(88.6)	6551	(78.9)
Yes	511	(31.6)	452	(21.5)	486	(19.9)	236	(11.0)	1685	(20.3)
Unknown	16	(1.0)	14	(0.7)	30	(1.2)	8	(0.4)	68	(0.8)
Co-infections, positive/tested										
HIV	48/1586	(3.0)	279/2075	(13.4)	215/2362	(9.1)	164/2008	(8.2)	706/8031	(8.8)
Syphilis	6/956	(0.6)	11/538	(2.0)	7/736	(1.0)	22/1108	(2.0)	46/3338	(1.4)
Hepatitis B	0/400	(0.0)	0/91	(0.0)	0/41	(0.0)	13/441	(2.9)	13/973	(1.3)
Malaria	190/1616	(11.8)	18/512	(3.5)	6/493	(1.2)	27/464	(5.8)	241/3085	(7.8)
COVID-19 risk behaviors^§^	n = 988		n = 1382		n = 1690		n = 1334		N = 5394	
Mask use in public all or most of the time (self-report)	428	(43.3)	398	(28.8)	755	(44.7)	1090	(81.7)	2671	(49.5)
Proper mask use during ANC visit	574	(58.1)	429	(31.0)	873	(51.7)	783	(58.7)	2659	(49.3)
Travel outside district	41	(4.1)	47	(3.4)	93	(5.5)	100	(7.5)	281	(5.2)
Travel outside Zambia	6	(0.6)	11	(0.8)	5	(0.3)	16	(1.2)	38	(0.7)
Visit to church or mosque	544	(55.1)	993	(71.9)	1201	(71.1)	886	(66.4)	3624	(67.2)
Visit to wedding or funeral	236	(23.9)	698	(50.5)	381	(22.5)	407	(30.5)	1722	(31.9)
Visit to market or grocery store	355	(35.9)	1014	(73.4)	1136	(67.2)	1081	(81.0)	3586	(66.5)
Indoor dining	64	(6.5)	437	(31.6)	250	(14.8)	352	(26.4)	1103	(20.4)
Use of public transportation	77	(7.8)	918	(66.4)	792	(46.9)	989	(74.1)	2776	(51.5)

ANC = antenatal care, IQR = interquartile range.

^†^Close contact defined as being within 2 meters of someone outside the household suspected or confirmed to have COVID-19 for greater than 15 minutes

^‡^≥1 dose of any COVID-19 vaccine

^§^Responses restricted to February-September 2022

**Fig 1:**
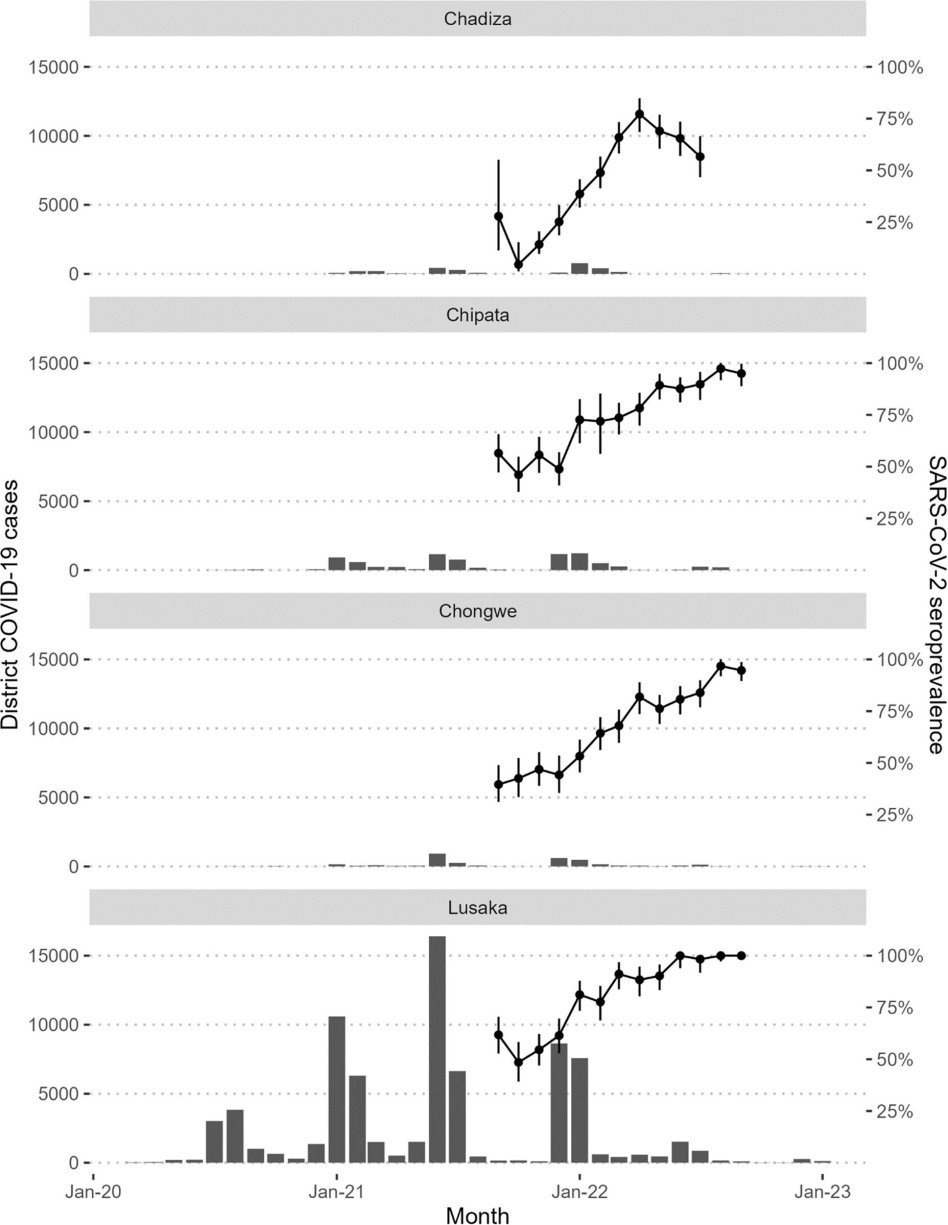
Reported COVID-19 cases among the general population and SARS-CoV-2 seroprevalence among study participants, by district. The bars represent COVID-19 case among all persons in the district reported by Zambia National Public Health Institute and the lines represent the monthly SARS-CoV-2 seroprevalence among pregnant women attending antenatal care in the district during the study. Serologic testing was done using the Tetracore FlexImmArray SARS-CoV-2 Human IgG Antibody Test; a positive result was assigned if signal ratios ≥1.2 for all 3 assay targets (nucleocapsid [N], receptor-binding domain [RBD] of the spike protein, and N-RBD fusion). Seroprevalence was adjusted for assay performance (sensitivity = 89.8% and specificity = 100%) and error bars represent 95% confidence intervals.

Overall, 5,296 (63.8%) participants were SARS-CoV-2 seropositive from September 2021 through September 2022. Seroprevalence was highest in Lusaka and lowest in Chadiza throughout the study period (Fig 1). In Lusaka, SARS-CoV-2 adjusted seroprevalence rose from 61.8% (95% confidence interval [CI]: 52.8–70.5%) in September 2021 to 100.0% in August and September 2022 (August 95% CI: 97.2–100.0; September 95% CI: 100.0–100.0). SARS-CoV-2 seroprevalence also peaked near 100% in August 2022 in Chipata (97.3% [91.7–100.0]) and Chongwe (96.8% [91.9–100.0]) districts but dropped slightly in September 2022 (95.1% [89.0–99.7] and 94.7% [89.6–98.8], respectively). In Chadiza, SARS-CoV-2 seroprevalence peaked at 77.2% (95% CI: 61.8–78.6) in April 2022 and then gradually dropped to 56.6% (95% CI: 46.7–66.5) when data collection ended in July 2022. The greatest single month increase in urban districts (Lusaka and Chipata) was from December 2021 to January 2022 during the peak of the Omicron wave in Zambia, while in rural districts (Chadiza and Chongwe), the greatest increases were seen in March and April 2022, respectively.

**Fig 2:**
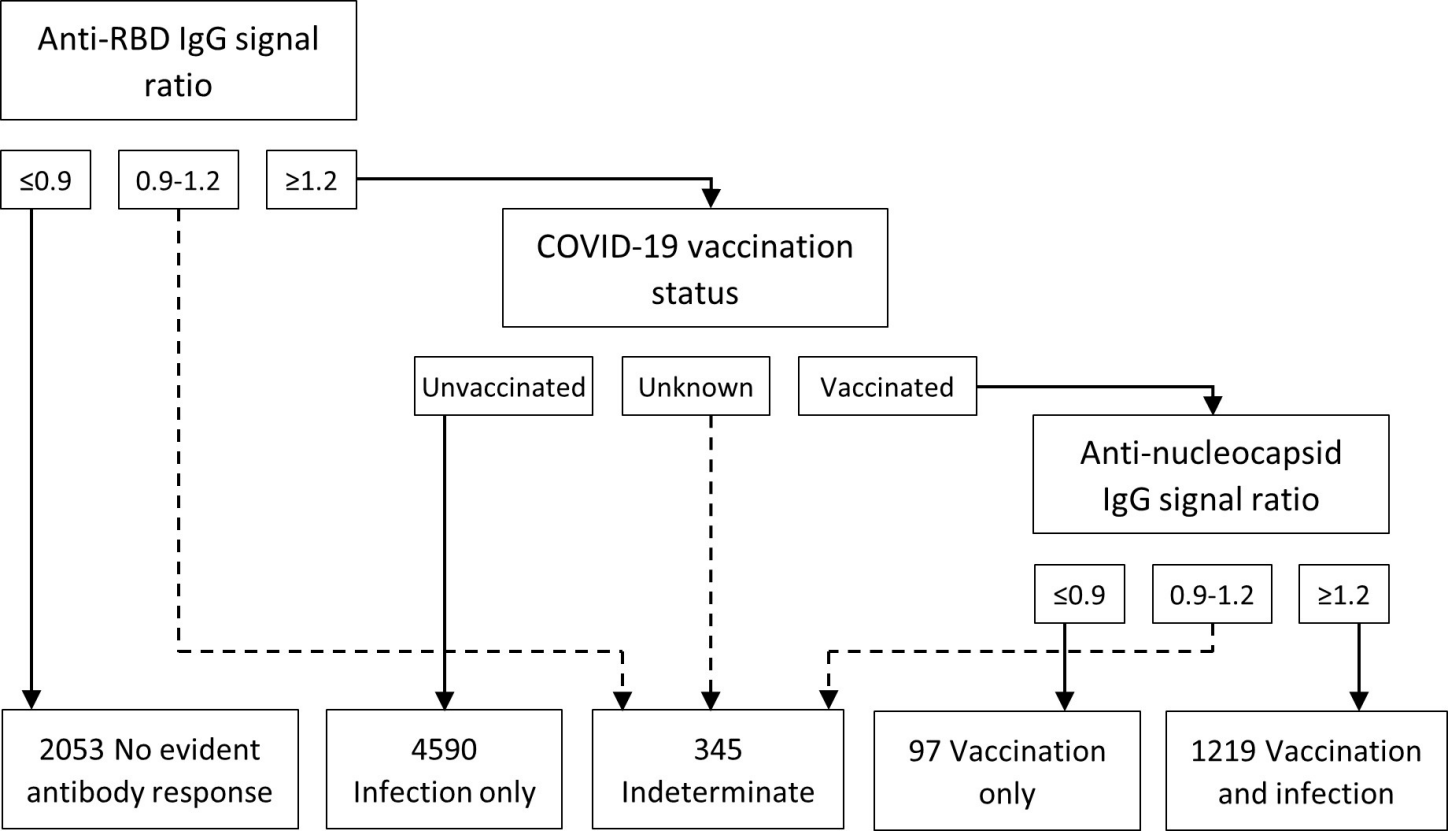
Antibody response categorization based on anti-RBD IgG, anti-nucleocapsid IgG, and COVID-19 vaccination status (N = 8,304). The Tetracore FlexImmArray SARS-CoV-2 Human IgG Antibody Test used is a multiplex bead assay with three SARS-CoV-2 targets (i.e., nucleocapsid, receptor-binding domain (RBD) of spike, and fusion) to facilitate distinguishing between infection only, vaccination only, and combined vaccination and infection antibody responses when considered with vaccination history. Participants who reported receiving a Sinopharm or unknown vaccine were categorized as indeterminate due to the inability to distinguish between vaccination only and hybrid vaccination and infection antibody responses from inactivated or attenuated virus vaccines. Adapted from Duarte et al. 2021.

**Fig 3:**
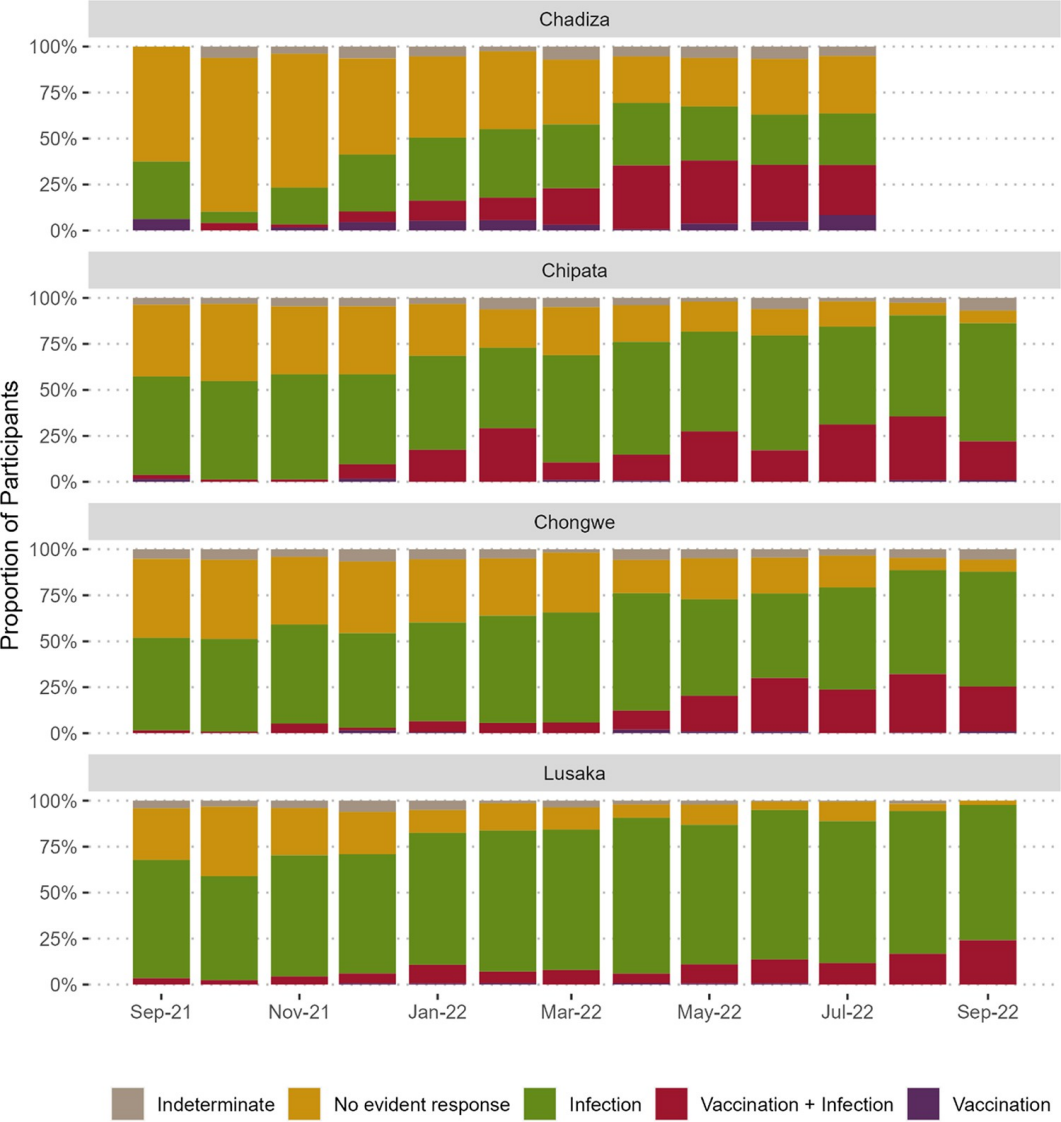
SARS-CoV-2 antibody response categorization of study participants by enrollment month and district. No evident antibody response was defined as testing negative for anti-RBD IgG. Infection-derived antibody response was defined as positive anti-RBD IgG AND no reported COVID-19 vaccination. Vaccination and infection-derived hybrid antibody response was defined as positive anti-RBD IgG AND positive anti-nucleocapsid IgG AND reported COVID-19 vaccination. Vaccination-derived antibody response was defined as positive anti-RBD IgG AND reported COVID-19 vaccination AND negative anti-nucleocapsid IgG. Equivocal IgG responses, unknown COVID-19 vaccination status, and unknown or Sinopharm vaccine type were categorized as indeterminate.

**Fig 4:**
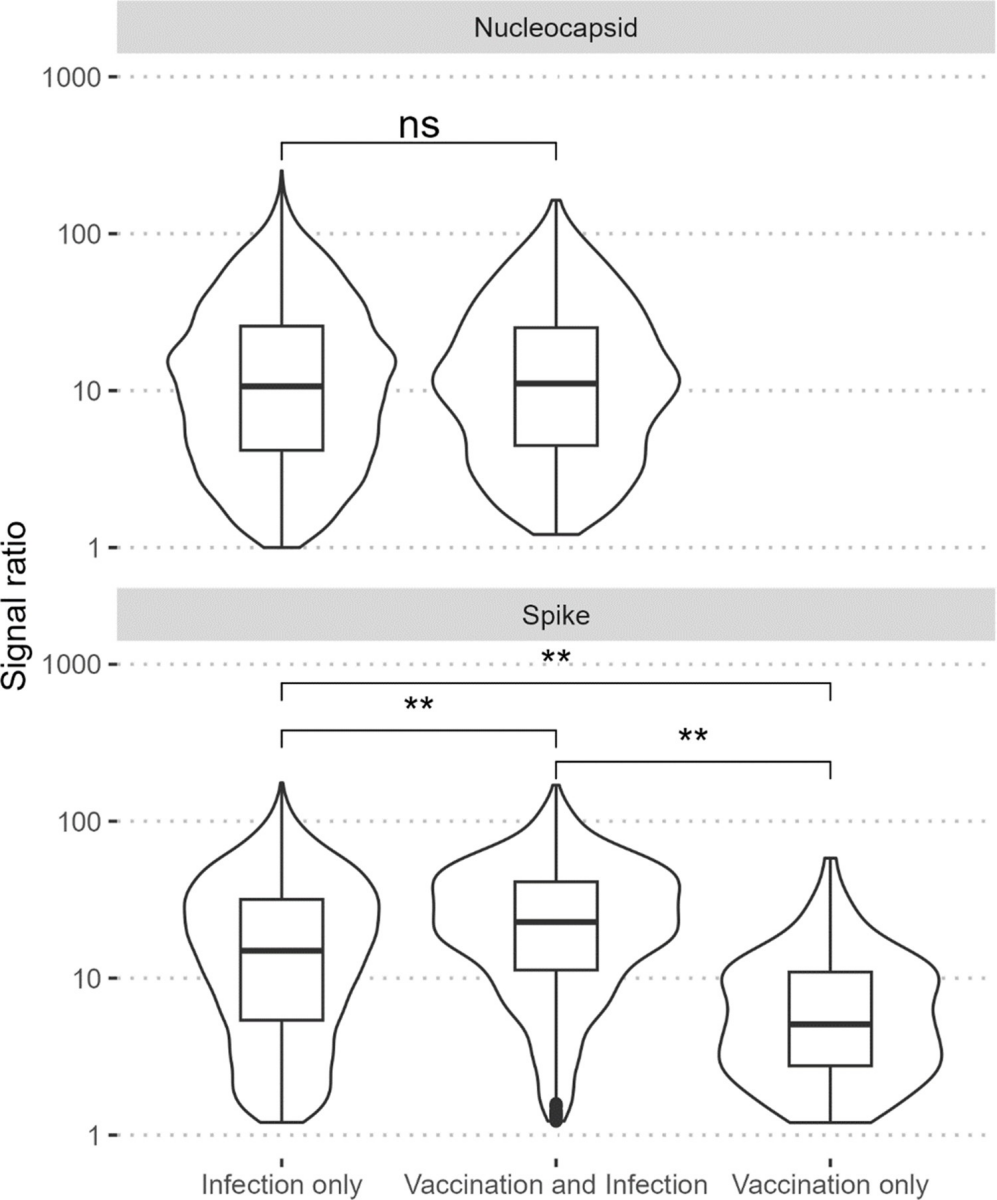
Signal ratios for three assay targets by antibody response category. Signal ratios were calculated by dividing the mean fluorescent intensity of the target by the mean fluorescent intensity of the calibrators. Vaccination and infection combined produced the strongest antibody response against all three assay targets compared to infection or vaccination alone. Participants whose antibody response was derived from infection only had greater signal ratios for the RBD target compared to participants with vaccination-derived antibody response only. ns = not significant (p-value>0.05); **p-value<0.001.

In the sub-analysis exploring target-specific assay results and COVID-19 vaccination status, 6,042 (72.8%) participants were positive for anti-RBD IgG and 6,343 (76.4%) were positive for anti-N IgG from September 2021 through September 2022. Overall, 2,053 (24.7%) participants had no detectible antibodies to the RBD or nucleocapsid proteins, and 345 (4.2%) were indeterminate (Fig 2). Among the remaining 5,906 participants with anti-RBD antibodies and a valid COVID-19 vaccination status, most (77.7%) were likely infection-induced only, 20.6% from vaccination and infection, and only 1.6% vaccination-induced alone. Infection-induced antibodies were the most common throughout the study period while hybrid infection and vaccination antibody responses became more common over time (Fig 3). In Chadiza, hybrid antibodies surpassed infection-induced starting in April 2022. In comparing target-specific signal ratios by antibody response category, no difference was observed for the nucleocapsid target (Fig 4). For the RBD target, the median signal ratio was significantly higher among participants who were vaccinated and infected compared to participants who were infected or vaccinated alone, and the median signal ratio for participants who were only infected was greater than for those who were only vaccinated.

Pairwise logistic regression adjusting for enrollment month identified different factors associated with SARS-CoV-2 serostatus in each district (Table 2). Women aged 40–49 years in Chipata had nearly three times the odds of seropositivity compared to women aged 15–19 years (aOR: 2.76 [95% CI: 1.16–6.58]), but age was not significantly associated with serostatus elsewhere. In Chadiza and Chongwe districts, women who had completed secondary education or higher had greater odds of SARS-CoV-2 seropositivity compared to women with some primary education (Chadiza aOR: 1.76 [1.17–2.64]; Chongwe aOR: 1.64 [1.16–2.31]). Significant differences were observed in SARS-CoV-2 seroprevalence by occupation in all districts except Chadiza. In Chipata, women living with HIV (WLHIV) had higher odds of seropositivity compared to HIV-negative women (aOR: 1.42 [1.05–1.90]), although this association was no longer significant after further adjusting for age group (aOR: 1.29 [0.95–1.76]).

**Table 2:** SARS-CoV-2 seropositivity by participant demographics and SARS-CoV-2 exposures and risk behaviors.

	Chadiza				Chipata				Chongwe				Lusaka			
	SARS-CoV-2 Seropositive		Adjusted Odds Ratio		SARS-CoV-2 Seropositive		Adjusted Odds Ratio		SARS-CoV-2 Seropositive		Adjusted Odds Ratio		SARS-CoV-2 Seropositive		Adjusted Odds Ratio	
	n	(%)	aOR	95% CI	n	(%)	aOR	95% CI	n	(%)	aOR	95% CI	n	(%)	aOR	95% CI
Total	707	(43.8)			1433	(68.3)			1565	(64.1)			1591	(74.1)		
Age																
15–19	182	(41.6)	1	(ref)	244	(66.1)	1	(ref)	330	(63.7)	1	(ref)	154	(73.3)	1	(ref)
20–29	343	(45.1)	1.23	(0.96–1.58)	808	(67.9)	1.12	(0.86–1.46)	823	(63.3)	1.00	(0.80–1.25)	947	(73.0)	0.98	(0.69–1.40)
30–39	114	(45.4)	1.12	(0.80–1.56)	338	(70.3)	1.31	(0.96–1.79)	352	(66.3)	1.21	(0.92–1.59)	443	(76.9)	1.22	(0.83–1.80)
40–49	12	(46.2)	1.43	(0.61–3.33)	36	(83.7)	2.76	(1.16–6.58)*	60	(66.7)	1.17	(0.70–1.95)	42	(73.7)	0.98	(0.48–2.00)
Education																
No education	156	(41.4)	0.91	(0.70–1.18)	108	(63.2)	0.86	(0.59–1.26)	44	(57.1)	1.17	(0.70–1.95)	16	(51.6)	0.44	(0.19–1.00)
Some primary	337	(43.3)	1	(ref)	329	(63.5)	1	(ref)	485	(60.0)	1	(ref)	296	(71.7)	1	(ref)
Completed primary	49	(36.8)	0.96	(0.64–1.43)	390	(70.5)	1.08	(0.82–1.41)	405	(66.6)	1.12	(0.89–1.42)	255	(75.7)	0.91	(0.64–1.30)
Some secondary	95	(47.5)	1.23	(0.88–1.70)	440	(71.9)	1.25	(0.96–1.63)	485	(66.3)	1.34	(1.07–1.67)	602	(74.8)	0.94	(0.70–1.25)
Completed secondary or higher	68	(55.3)	1.76	(1.17–2.64)*	161	(67.1)	1.14	(0.81–1.61)	146	(67.9)	1.64	(1.16–2.31)*	420	(75.1)	0.96	(0.71–1.31)
Occupation																
Farmer	596	(43.1)	0.63	(0.37–1.10)	274	(61.9)	0.66	(0.51–0.85)*	349	(58.0)	0.70	(0.56–0.87)*	9	(90.0)	2.55	(0.30–21.85)
Daily labourer/pieceworker	4	(50.0)	1.20	(0.25–5.81)	49	(62.8)	0.76	(0.46–1.26)	205	(63.1)	0.88	(0.67–1.16)	55	(85.9)	1.93	(0.90–4.15)
Merchant/shop owner	3	(25.0)	0.25	(0.06–1.08)	30	(53.6)	0.55	(0.31–0.98)*	33	(60.0)	1.08	(0.6–1.95)	83	(76.9)	1.94	(1.19–3.18)*
Health worker	4	(50.0)	1.11	(0.24–5.10)	8	(50.0)	0.50	(0.17–1.42)	5	(83.3)	4.70	(0.48–45.97)	10	(76.9)	1.60	(0.40–6.40)
Government/civil servant	13	(48.1)	0.83	(0.32–2.19)	35	(74.5)	1.16	(0.58–2.33)	15	(88.2)	5.02	(1.07–23.47)*	61	(69.3)	0.91	(0.55–1.52)
Private sector employee	3	(50.0)	0.66	(0.11–3.95)	21	(67.7)	0.91	(0.41–2.02)	20	(80.0)	3.38	(1.19–9.58)*	107	(73.8)	1.11	(0.73–1.70)
Hawker/market seller	6	(42.9)	1.05	(0.30–3.67)	102	(71.8)	1.00	(0.67–1.51)	121	(73.8)	1.56	(1.05–2.34)*	181	(75.1)	1.20	(0.86–1.69)
Student	42	(47.2)	0.61	(0.30–1.23)	70	(73.7)	1.16	(0.70–1.91)	54	(68.4)	1.15	(0.69–1.94)	88	(79.3)	0.94	(0.56–1.58)
Unemployed	34	(54.0)	1	(ref)	719	(72.2)	1	(ref)	712	(65.4)	1	(ref)	859	(71.9)	1	(ref)
Other occupation	0	(0.0)	--	--	102	(63.0)	0.76	(0.53–1.10)	37	(68.5)	1.24	(0.66–2.33)	129	(79.6)	1.34	(0.87–2.06)
Prior COVID-19																
No	678	(43.3)	1	(ref)	1389	(68.4)	1	(ref)	1525	(64.2)	1	(ref)	1518	(73.9)	1	(ref)
Yes	28	(66.7)	3.06	(1.55–6.07)*	28	(66.7)	0.95	(0.48–1.89)	22	(71.0)	1.68	(0.72–2.95)	52	(81.2)	1.25	(0.64–2.45)
Household COVID-19																
No	684	(43.5)	1	(ref)	1379	(68.5)	1	(ref)	1451	(63.8)	1	(ref)	1458	(73.5)	1	(ref)
Yes	17	(47.2)	1.20	(0.60–2.38)	31	(63.3)	0.95	(0.51–1.77)	36	(75.0)	1.45	(0.71–2.95)	112	(81.8)	1.19	(0.75–1.91)
Close COVID-19 contact^†^																
No	629	(43.7)	1	(ref)	1348	(69.0)	1	(ref)	1277	(64.1)	1	(ref)	1365	(73.3)	1	(ref)
Yes	11	(36.7)	0.93	(0.42–2.05)	32	(61.5)	0.80	(0.44–1.46)	41	(66.1)	0.95	(0.53–1.67)	140	(79.5)	1.10	(0.73–1.66)
COVID-19 vaccination^‡^																
No	396	(36.4)	1	(ref)	1057	(64.7)	1	(ref)	1164	(60.5)	1	(ref)	1379	(72.4)	1	(ref)
Yes	299	(58.5)	1.76	(1.40–2.21)*	363	(80.3)	1.48	(1.13–1.93)*	381	(78.4)	1.40	(1.09–1.80)*	208	(88.1)	1.97	(1.29–3.02)*
HIV status																
Negative	767	(44.0)	1	(ref)	1212	(67.5)	1	(ref)	1404	(65.4)	1	(ref)	1388	(75.3)	1	(ref)
Positive	16	(33.3)	0.83	(0.43–1.59)	203	(72.8)	1.42	(1.05–1.90)*	113	(52.6)	0.77	(0.57–1.05)	104	(63.4)	0.87	(0.60–1.26)

aOR = adjusted odds ratio (adjusted for enrollment month); CI = confidence interval.

^†^Close contact defined as being within 2 meters of someone outside the household suspected or confirmed to have COVID-19 for greater than 15 minutes

^‡^≥1 dose of any COVID-19 vaccine.

*p-value<0.05

SARS-CoV-2 seropositivity was significantly associated with prior confirmed COVID-19 only in Chadiza (aOR = 3.06 [1.55–6.07]; Table 2). Among 179 reported prior COVID-19 cases, 130 (72.6%) were SARS-CoV-2 seropositive at the time of enrollment. The median time between COVID-19 diagnosis and first ANC visit was 9 months (IQR: 7–14) among women who tested SARS-CoV-2 seropositive and 8 months (IQR: 4–13) among seronegative women (p = 0.13).

COVID-19 vaccination was associated with significantly higher odds of SARS-CoV-2 seropositivity in all four districts, adjusting for enrollment month (Table 2). Among vaccinated women, SARS-CoV-2 serostatus did not differ by partial vs. full primary vaccine series or vaccine type (Table 3). Compared to women who received their first COVID-19 vaccine dose within one month prior to study enrollment, participants who received their first dose one to five months prior had greater odds of testing SARS-CoV-2 seropositive, controlling for enrollment month and district (aOR: 1.58 [1.19–2.10]).

**Table 3:** SARS-CoV-2 seroprevalence among vaccinated participants (n = 1685).

	SARS-CoV-2 Seropositive		SARS-CoV-2 Seronegative		Adjusted Odds Ratio	
	n	(%)	n	(%)	aOR	(95% CI)
Vaccination status						
Partially vaccinated	123	(70.3)	52	(29.7)	0.74	(0.51–1.07)
Full primary series	1106	(74.9)	370	(25.1)	1	(ref)
Vaccine type						
Oxford/AstraZeneca (ChAdOx1-S)	150	(64.9)	81	(35.1)	1	(ref)
J&J/Janssen (Ad.26.COV2.S)	965	(75.7)	309	(24.3)	1.28	(0.92–1.79)
Pfizer-BioNTech (BNT162b2)	50	(75.8)	16	(24.2)	0.71	(0.36–1.39)
Sinopharm	32	(72.7)	12	(27.3)	0.79	(0.37–1.69)
Mixed	21	(91.3)	2	(8.7)	2.82	(0.60–13.26)
Other	7	(100.0)	0	(0.0)	--	--
Months between first dose and enrollment						
<1 month	290	(69.5)	127	(30.5)	1	(ref)
1–5 months	669	(75.8)	214	(24.2)	1.58	(1.19–2.10)*
≥6 months	223	(81.4)	51	(18.6)	1.48	(0.99–2.22)

aOR = adjusted odds ratio (adjusted for district and enrollment month); CI = confidence interval.

*p<0.05

Twenty-one percent of the 179 participants with prior COVID-19 reported being hospitalized. Hospitalization and time between COVID-19 and enrollment were not associated with SARS-CoV-2 serostatus, but COVID-19 vaccination was associated with greater odds of seropositivity among these women (OR: 4.32 [2.07–9.02]; Table 4).

**Table 4:** Characteristics participants reporting prior COVID-19 by SARS-CoV-2 serostatus (n = 179).

	SARS-CoV-2 Seropositive		SARS-CoV-2 Seronegative		Adjusted Odds Ratio	
	n	(%)	n	(%)	aOR	(95% CI)
Hospitalized due to COVID-19						
No	100	(78.7)	39	(79.6)	1	(ref)
Yes	27	(21.3)	10	(20.4)	0.63	(0.24–1.66)
Months between diagnosis and enrollment						
<6 months	20	(18.3)	13	(31.0)	1	(ref)
6–11 months	41	(37.6)	12	(28.6)	1.02	(0.37–2.79)
≥12 months	48	(44.0)	17	(40.5)	0.41	(0.11–1.51)
COVID-19 vaccination^†^						
No	54	(41.9)	37	(75.5)	1	(ref)
Yes	75	(58.1)	12	(24.5)	4.11	(1.80–9.41)*

aOR = adjusted odds ratio (adjusted for district and enrollment month); CI = confidence interval

^†^Received ≥1 doses of any COVID-19 vaccine.

*p<0.05

District-level cumulative incidence was calculated from 2022 population projections and reported COVID-19 cases from March 2020 through the end of the survey (July 2022 for Chadiza and September 2022 elsewhere) and ranged from 1.1% in Chadiza District to 3.5% in Lusaka District (Table 5). Overall, 90,150 COVID-19 cases were reported in the four districts from March 2020 to September 2022 but we estimated that 2,911,393 people had evidence of SARS-CoV-2 exposure. The ratio of SARS-CoV-2 seroprevalence to cumulative incidence was 29:1 in Lusaka District, 31:1 in Chadiza District, 39:1 in Chipata District, and 92:1 in Chongwe District.

**Table 5:** Estimated populations exposed to SARS-CoV-2 and ratios of peak seroprevalence to cumulative incidence during last survey month.

	Chadiza	Chipata	Chongwe	Lusaka
Peak SARS-CoV-2 seroprevalence^†^	77.2%	97.3%	96.8%	100.0%
2022 census population	111,069	327,059	313,389	2,204,059
Reported COVID-19 cases	2,761	8,096	3,303	75,990
Cumulative incidence^‡^	2.5%	2.5%	1.1%	3.4%
Estimated population with evidence of SARS-CoV-2 exposure^§^	85,745	318,228	303,361	2,204,059
Ratio of seroprevalence to cumulative incidence	31:1	39:1	92:1	29:1

^†^Peak SARS-CoV-2 seroprevalence for Chadiza District was April 2022; for Chipata District, peak was August 2022; for Chongwe district, peak was August 2022; for Lusaka District, peak was September 2022.

^‡^Calculated as reported cases divided by population projection.

^§^Calculated as seroprevalence multiplied by population projection. 2022 census data were obtained from the Zambia Statistics Agency and reported COVID-19 cases were obtained from the Zambia National Public Health Institute.

## Discussion

SARS-CoV-2 seroprevalence was high among participants and nearly all first ANC attendees in three out of four study districts had evidence of SARS-CoV-2 infection by September 2022. In those districts, seroprevalence generally increased over the 13-month study period, while in Chadiza, it declined from April to July 2022. Lower seroprevalence in Chadiza District was expected, being the most rural of the study districts. The very high seroprevalence in Lusaka District was also not surprising given a study conducted at two Lusaka hospitals which found that 36.9% of women attending ANC clinics between March and July 2021 (part of the Delta wave, prior to this study) were positive for SARS-CoV-2 by RT-PCR [23]. The large rise in SARS-CoV-2 seroprevalence from November 2021 to March 2022 when the Omicron wave occurred was similar to what was seen in South Africa where seroprevalence among the general population was 73% before the Omicron wave and 91% after [24, 25]. This high seroprevalence was proposed as a reason for the lack of significant transmission waves after Omicron, despite the emergence of Omicron sub-lineages with greater infectivity and low COVID-19 vaccine coverage [25].

COVID-19 vaccination among participants was low and most SARS-CoV-2 antibodies were likely infection-induced. COVID-19 vaccination was associated with higher odds of SARS-CoV-2 seropositivity and COVID-19 vaccination in combination with SARS-CoV-2 infection produced the strongest immune response, both of which could be explained by a robust immune response caused by hybrid immunity [26]. Other studies in Africa also measured higher SARS-CoV-2 seroprevalence among vaccinated participants, higher neutralizing antibody titers among participants with infection and vaccination, and faster waning of antibodies post-vaccination among participants who had not been infected [7, 27–29]. These results support the recommendation for COVID-19 vaccination (including booster doses) regardless of past infection. COVID-19 vaccination campaigns were conducted throughout Zambia during the study period, but vaccine coverage among study participants was lower than the general population, possibly reflecting hesitancy toward vaccination during pregnancy, and only about 12% of the fully vaccinated population has received a booster dose [2]. COVID-19 messages and risk mitigation strategies addressing the concerns of pregnant women can be incorporated seamlessly into routine ANC to prevent adverse outcomes in this population at higher risk for severe COVID-19. Healthcare providers can screen for symptoms of COVID-19, malaria, and tuberculosis simultaneously, and COVID-19 vaccines may be administered alongside tetanus boosters.

The ratios of SARS-CoV-2 seroprevalence to cumulative incidence were lower than the 1:92 ratio previously measured in a July 2020 serosurvey in other parts of Zambia [3]. This reduction mirrors the downward global trend in seroprevalence to cumulative incidence ratios since 2020 (i.e., improving case detection ratios) and may be due to evolving testing strategies and increased availability and use of rapid antigen test kits [30]. Interpretation of these ratios late in the pandemic is complicated by reinfections, waning antibodies, and non-report of home RDT results, leading to an underestimation of total SARS-CoV-2 infections. Some past SARS-CoV-2 infections may not be detectable as antibodies wane over time and people with asymptomatic infections or mild clinical cases may mount less of an immune response [31]. This is consistent with the women who were SARS-CoV-2 seronegative despite reporting prior COVID-19 and may explain the declining seroprevalence observed in some districts and months.

Older age, higher education, and urban versus rural location were all associated with higher seroprevalence during the July 2020 survey in Zambia, and similar associations were noted in some districts during this study [3]. This may reflect differences in access to COVID-19 information and infection prevention supplies or in the ability to comply with COVID-19 risk mitigation guidelines among certain subpopulations. These factors may intersect with occupation which was also associated with differences in seroprevalence in most districts. Diverse work environments, such as open fields for farming, compact cubicles in an office, or crowded market stalls, could impact an individual’s SARS-CoV-2 exposure risk. Similar associations were noted in Mozambique where market sellers and those engaged in formal employment had higher SARS-CoV-2 seroprevalence than other community members [29]. Furthermore, modes of transportation might vary by urban/rural status, which could in turn affect risk of coming into contact with SARS-CoV-2 virus. Tailored COVID-19 prevention messaging for demographic groups with higher SARS-CoV-2 seroprevalence may help reduce transmission during future waves. These observed differences in SARS-CoV-2 prevalence by certain demographic groups could simply reflect differences in COVID-19 risk for different groups in the population.

Most COVID-19 mitigation strategies in Zambia were carried out nationally by the Ministry of Health. Given the differences in SARS-CoV-2 risk factors and seroprevalence trends by district, subnational strategies may be more suitable in the future to mitigate COVID-19 risk where and when needed. District or provincial level responses could help direct resources to areas of high transmission while avoiding imposing strict measures in areas with low transmission where the social and economic burdens of mitigation efforts may outweigh the benefit of reducing transmission. This approach was used to lift masking requirements in Zambia as districts reached 70% vaccination coverage and demonstrates that robust surveillance systems and quality tracking of key COVID-19 indicators at the district level can facilitate subnational decision-making [32].

This study has several limitations. First, we could not estimate a single population-weighted seroprevalence because of the non-systematic selection of districts and participants. Our study was limited to women aged 15–49 years and may not be representative of populations that may be more or less susceptible to SARS-CoV-2 infection. However, a global meta-regression analysis found no difference in seroprevalence by sampling frame comparing studies among pregnant women to household and community surveys [30]. As with all SARS-CoV-2 seroprevalence surveys, our estimates depend on serologic test performance. The manufacturer’s requirement of testing positive for all three targets for an overall positive sample is more conservative than single target assays and may have excluded some past infections. Waning of SARS-CoV-2 antibodies might have resulted in underestimation of seroprevalence, and variability in the waning time for different antibody types could have biased the sub-analysis findings categorizing infection/vaccination statuses. Studies have shown that anti-nucleocapsid antibodies wane more quickly than anti-spike antibodies [33, 34], although anti-N antibody prevalence was slightly greater than anti-RBD antibody prevalence in our study. Adjusting the seroprevalence estimates based on a test sensitivity of 90% likely helped but the potential for misclassification remains. Given the cross-sectional study design, we could not assess whether those who were seronegative had truly never been exposed to SARS-CoV-2, had been exposed and never mounted a detectable antibody response, or had sero-reverted before testing. Lastly, we were uncertain how the premature ending of SARS-CoV-2 surveillance in Chadiza in July 2022 affected the overall seroprevalence estimates for this study.

Despite these limitations, our study also has notable strengths. We measured SARS-CoV-2 seroprevalence over 13 months which encompassed periods before, during, and after Zambia’s Omicron wave. The study period also spanned early COVID-19 vaccine rollout in Zambia, and the use of a multiplex bead assay with spike and nucleocapsid targets allowed us to distinguish seropositivity from infection versus vaccination. We included several districts which had not been included in previous SARS-CoV-2 seroprevalence studies and found substantial geographic variation, particularly in the most rural study district. Finally, we demonstrated the feasibility of integrating SARS-CoV-2 serosurveillance into routine health services which may be adapted in future epidemics.

As the COVID-19 pandemic continues to evolve, so does the role of seroprevalence studies. While our study shows that most people living in Zambia have likely been exposed to SARS-CoV-2, seroprevalence studies may still play a valuable role in understanding waning population immunity over time and susceptibility to emerging variants. Longitudinal cohort studies may be better suited than cross-sectional surveys to meet these needs, and ANC may continue to be a useful platform for recruiting participants as women attend frequent follow-up visits during pregnancy and postpartum periods.

## Supporting information

S1 Checklist Inclusivity in global research. (DOCX)
